# Macrophage Plasma Membrane Coating Enhances Nerve Delivery of Stably Labeled Polymeric Nanoparticles

**DOI:** 10.1021/acsnanomed.6c00218

**Published:** 2026-10-07

**Authors:** Chanpreet Kaur, Maleen H. Cabe, Charles J. Kasella, Natalie Velazquez, Kelly A. Langert

**Affiliations:** † Department of Molecular Pharmacology and Neuroscience, Loyola University Chicago, Stritch School of Medicine, Maywood, Illinois 60153, United States; ‡ Research Service, Edward Hines Jr. VA Hospital, Hines, Illinois 60141, United States

**Keywords:** peripheral nerve, targeted delivery, nanomedicine, inflammation, neuropathy

## Abstract

Acute inflammatory demyelinating polyneuropathy (AIDP) is the leading cause of acute autoimmune flaccid paralysis and the most common subtype of Guillain–Barré syndrome (GBS). A key pathogenic event in AIDP is localized inflammation of the blood–nerve barrier (BNB), which enables transendothelial migration of circulating autoreactive leukocytes into peripheral nerves. Experimental autoimmune neuritis (EAN), a well-established rat model of GBS, has provided critical insights into AIDP pathophysiology; however, therapeutic options remain limited, in part due to the restrictive BNB. Nanomedicine offers a unique approach to deliver a therapeutic payload across the BNB and to nerves while minimizing off-target effects and systemic toxicity. We previously found that EAN-associated BNB permeability promotes passive accumulation of long-circulating nanoparticles (NPs) in inflamed nerves at advanced disease stages. While promising, we hypothesize that surface modification with ligands that target the underlying pathology can enhance site-specific targeting and improve NP delivery to EAN nerves. Here, we developed a NP-based drug delivery system using rat macrophage-derived plasma membrane vesicles (mNPs) to target affected peripheral nerves in EAN, leveraging the inflammation-driven pathology of the disease. We demonstrate that mNPs exhibit enhanced specificity and accumulation within inflamed nerves at both early and advanced stages of EAN. Interaction with the BNB is intercellular adhesion molecule-1 (ICAM-1)-dependent and leads to association with endoneurial cellular targets, including myelin-associated glia. These findings highlight the potential of macrophage-mimetic NPs as a platform for targeted therapeutic delivery and support their use as a vector for repurposing promising therapeutics for immune-mediated neuropathies.

## Graphical abstract

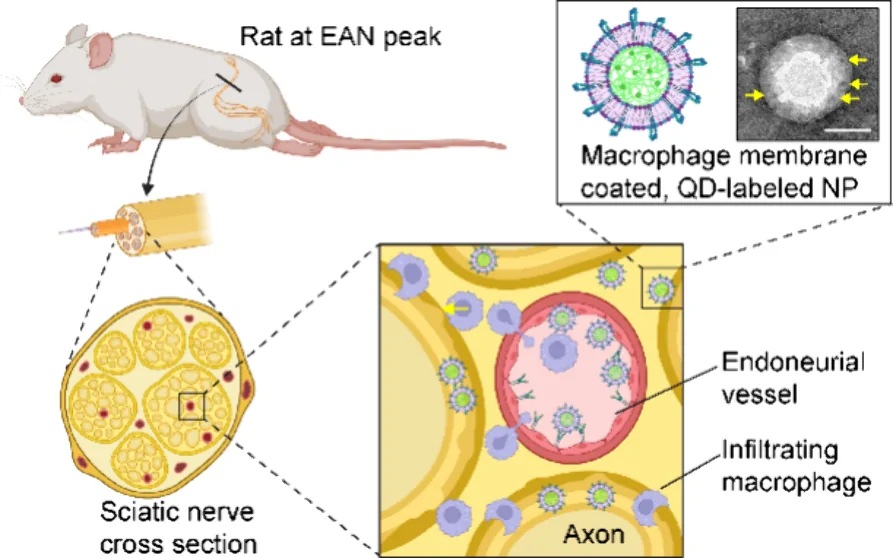

Acute inflammatory demyelinating polyneuropathy (AIDP), the most common subtype of Guillain-Barré syndrome (GBS), is a debilitating autoimmune disorder that affects the peripheral nervous system. GBS is the leading cause of acute flaccid paralysis worldwide, with an estimated annual incidence of approximately 100,000.^1^ Current treatment strategies, including intravenous immunoglobulin (IVIg) and plasma exchange, are costly, nonspecific, and not always effective, highlighting a critical need for more efficient disease-modifying therapeutic strategies.^2–4^ The well-established experimental autoimmune neuritis (EAN) model of AIDP has been instrumental in identifying immune mechanisms that drive peripheral nerve injury.^5–7^ With this model, a host of preclinical studies have demonstrated the potential of several different anti-inflammatory and immunomodulatory agents to therapeutically attenuate disease.^6,8–13^ Clinical translation of these findings remains hindered by the presence of the blood-nerve barrier (BNB) and the anatomical restriction of the peripheral nerves.^14^ As a result, achieving therapeutic concentrations often requires significantly elevated systemic doses, thereby increasing the risk of adverse off target effects and limiting clinical applicability.

Nanomedicine offers an emerging approach for effective delivery of therapeutics to sites of inflammation while minimizing systemic toxicity and off-target effects. Long circulating nanoparticles (NPs) can passively accumulate at inflamed or injured sites through an enhanced permeation and retention (EPR)-like effect, even within the nervous system.^15–17^ We previously showed that EAN-associated BNB permeability enables accumulation of poly­(ethylene)­glycol (PEG)-functionalized NPs in inflamed nerves during intermediate and peak stages of disease.^18^ While PEGylation can reduce opsonization, prolong system circulation and reduce aggregation, it does not confer cell-type specificity, and is still ultimately subject to immune clearance.^19,20^ Further, while PEGylated NPs entered nerves at peak disease, an ideal nanomedicine therapeutic would have the greatest therapeutic impact if administered earlier in disease.^21^

Surface modification with targeting ligands can enhance site-specific targeting of circulating NP delivery systems.^22,23^ Immune cell plasma membranes are a source of material for surface functionalization that accomplishes site-specific targeting as well as additional benefits, including immune system evasion and increased circulation time.^20,22^ Previous studies have shown tissue targeting through coating of NPs with membranes isolated from an array of leukocytes, including neutrophils and T lymphocytes.^23,24^ These biomimetic strategies leverage ligand-integrin interactions to mediate adhesion and homing to activated endothelium. In particular, the interaction between lymphocyte function-associated antigen-1 (LFA-1) and intercellular adhesion molecule-1 (ICAM-1) robustly promotes *in vivo* NP targeting to inflamed endothelium^25,26^ and is also a well-characterized pathogenic signaling axis in EAN/AIDP.^5^

Here, we build upon our previous finding of passive targeting of PEGylated NPs to nerves at peak EAN by evaluating systemic administration of immune cell membrane-coated NPs during early to peak stages of disease. With this top-down biomimetic strategy, we aim to improve the timing and distribution of targeted NP accumulation by mimicking the natural trafficking behavior of the autoreactive leukocytes implicated in the pathogenesis of GBS.^5,6^ Furthermore, by utilizing macrophage-derived membranes enriched in LFA-1 and other β2 integrin heterodimers, we will promote active targeting to inflammation-associated molecules including ICAM-1, which is upregulated by BNB endothelial cells during early disease stages.^18^ This targeted delivery strategy holds the potential for therapeutic intervention before the peak stage of disease, potentially preventing respiratory paralysis, avoiding the need for mechanical ventilation, and reducing severe disability and mortality.^3^

In this study, we developed NPs coated with macrophage-derived plasma membrane vesicles (mNPs) as a targeted drug delivery system to enhance peripheral nerve delivery in EAN. We isolated plasma membranes from an intrinsically active rat macrophage cell line enriched with integrin targeting ligands,^27–30^ and we optimized parameters for unilamellar coating of vesicles onto stably labeled polymeric NPs. Our results demonstrate that mNPs exhibit increased specificity and accumulation in inflamed peripheral nerves early in EAN. *In vitro*, mNP association with BNB endothelial cells is ICAM-1 dependent. *In vivo*, mNPs associate with endoneurial cellular targets, including Schwann cells, highlighting their potential for targeted therapeutic delivery.

## Results

### Identifying Cell Sources for BNB Specificity *in Vitro*

To begin assessing active targeting to rat peripheral nerve vasculature, we first identified and validated appropriate cellular sources for plasma membrane isolation and *in vitro* modeling of the BNB. Using our previously established methods,^31^ we isolated and cultured primary peripheral nerve microvascular endothelial cells (PNMECs). To obtain purified endothelial cell cultures, we eliminated contaminating fibroblasts with complement-mediated cell lysis. In the absence of lysis, mixed cultures (Figure 1A, top row) consisted of primarily Thy1.1/CD90^+^ fibroblasts (red), while early purification with a Thy1.1-targeting antibody and rabbit complement (Figure 1B, bottom row) allowed for proliferation of CD31^+^ endothelial cells (green).

**Figure 1:**
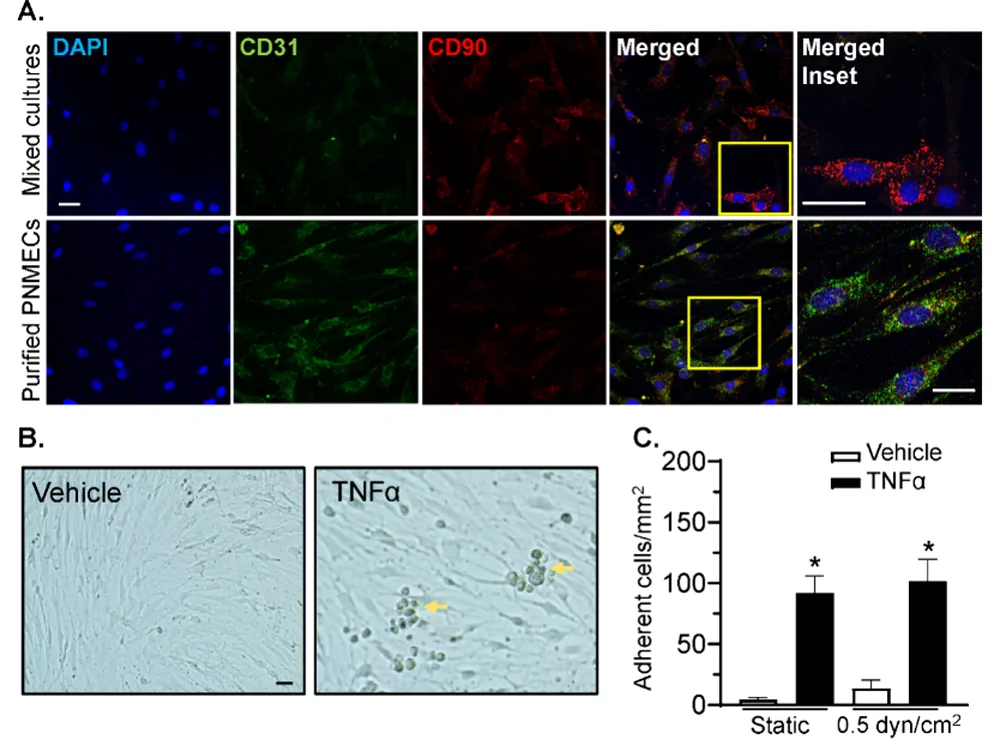
Identification of cellular sources for plasma membrane vesicles and *in vitro* BNB. (A) Representative confocal images of primary PNMEC cultures that were cultured without (top, “mixed cultures”) or with (bottom, “purified PNMECs”) exposure to Thy1.1 antibody-containing media. Red, CD90 (fibroblast marker); green, CD31 (endothelial cell marker). Yellow squares in merged images depict inset area. (B) Representative brightfield images of vehicle (left) and TNFα treated (right) PNMEC monolayers exposed to NR8383 macrophages. Yellow arrows indicate adherent macrophages. (C) Quantification of macrophage adhesion to vehicle or TNFα-treated primary PNMEC monolayers under static and sheer flow conditions, as indicated. Data shown are the mean ± SEM, *n* = 3 separate experiments. *p < 0.05 vs vehicle-treated control, ordinary one-way ANOVA with Tukey’s post hoc test. All scale bars, 30 μm.

It is well established that immune cells, including T cells and macrophages, cross the BNB and infiltrate nerves over the course of EAN.^32,33^ Compared with T cells, macrophages display a complete repertoire of the four classes of β2 integrins that mediate adhesion and migration.^34^ We hypothesized that the extensively characterized NR8383 rat alveolar macrophage cell line,^27–29^ which remains the only commercially available source of rat-derived myeloid cells,^30^ would enable specific binding to the inflamed BNB *in vitro* and *in vivo*. We quantified adhesion of unstimulated NR8383 rat alveolar macrophages to quiescent (vehicle treated) and inflamed (TNFα stimulated) PNMEC monolayers. Representative brightfield images (Figure 1B) depict binding of macrophages (yellow arrows) to inflamed PNMECs compared to quiescent PNMECs in a static adhesion assay. Under both static and physiologically relevant shear flow conditions, macrophage adhesion to quiescent monolayers was minimal (4.4 ± 1.77 and 13.7 ± 6.74 cells/mm^3^, respectively), while adhesion to inflamed monolayers was significantly increased (92.03 ± 13.89 and 101.97 ± 17.83 cells/mm^3^, respectively, *p* < 0.001) (Figure 1C). These results validate the selection of NR8383 cells as an intrinsically active,^27^ consistently renewable source of immune cells for membrane isolation.

### Isolation and Characterization of NR8383 Macrophage Plasma Membrane Vesicles

To isolate plasma membrane material, NR8383 macrophages were first gently lysed using nitrogen cavitation and then subjected to a series of differential centrifugation steps (Figure 2A). This workflow enabled stepwise removal of nuclei and debris, mitochondria, and other organelles, culminating in the enrichment of plasma membranes in the final pellet fraction. The protein profile of all fractions was assessed with Western immunoblotting (Figure 2B) after lyophilization and reconstitution. Plasma membrane associated proteins CD18, CD11b, and CD45 were enriched in the plasma membrane fraction (Lane III), even after lyophilization and reconstitution (Lane III*), particularly compared with the whole cell lysate (Lane V). Markers for other cellular compartments including histone (nuclear), apoptosis-inducing factor (AIF, mitochondrial), and GAPDH (cytosolic) were absent or significantly reduced in plasma membranes and enriched in each corresponding purified fraction (Lanes I, II, and IV, respectively).

**Figure 2:**
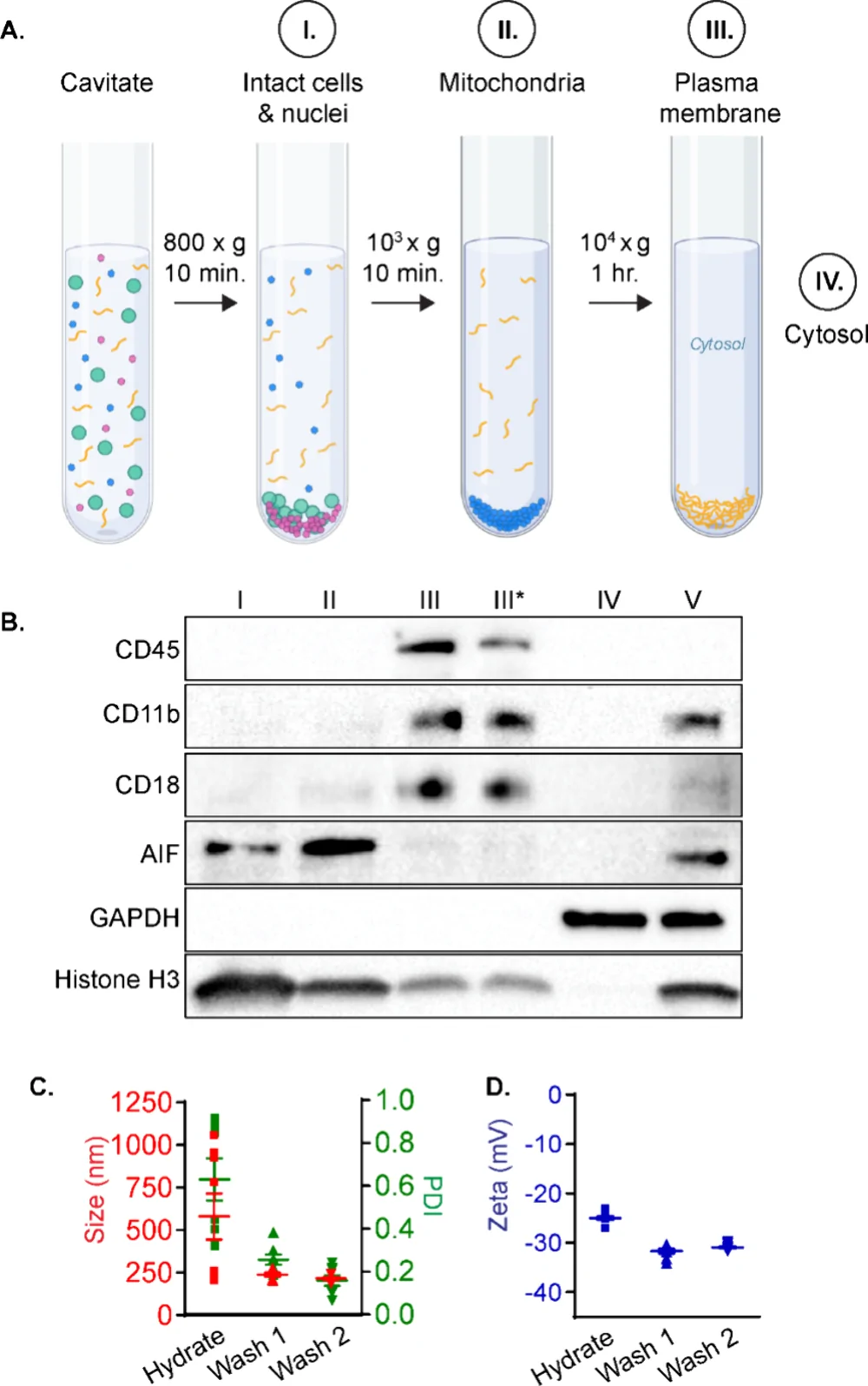
Isolation and characterization of rat macrophage plasma membrane vesicles. After rupture with nitrogen cavitation, NR8383 macrophage subcellular fractions, including nuclei (I), mitochondria (II), plasma membrane (III), and cytosol (IV), were collected using differential centrifugation (A). Fractions were identified using western immunoblot (B) with plasma membrane (CD18, CD11b, CD45), mitochondrial (AIF), nuclear (histone H3) and cytosolic (GAPDH) markers, and relative abundance of associated proteins was assessed in relation to whole cell lysate (V). Asterisk indicates lyophilized and reconstituted plasma membranes. Polydispersity index (PDI), size (C), and zeta potential (D) of lyophilized and reconstituted vesicles were determined before and after washing. Data shown are the mean ± SEM, *n* = 8 separate preparations.

Large, polydisperse vesicles were formed upon rehydrating lyophilized plasma membrane material (579.66 ± 135.2 nm with 0.63 ± 0.09 PDI, Figure 2C). Stepwise sonication and washing reduced vesicle size to 216.21 ± 9.63 nm with 0.15 ± 0.02 PDI (Figure 2C). Zeta potential measurements further confirmed the surface charge characteristics of the membrane vesicles, with values (−30 ± 0.22 mV) remaining within the expected range for negatively charged lipid bilayers throughout the processing steps (Figure 2D).^35^

### Quantum Dot Labeling of PLGA NP Cores

To facilitate tracking of NP fate in the microvasculature of the peripheral nerves *in vivo*, NP cores were labeled by covalently attaching spectrally defined (λ_ex_/λ_em_ = 445–460/525) quantum dots (QD_525_) using methods established by Medina et al. (Figure 3A).^36^ QD-conjugated NP cores exhibited a mean hydrodynamic diameter of 124.35 ± 2.2 nm, 0.07 ± 0.008 PDI, and a zeta potential of −16.7 ± 0.6 mV. These parameters are similar to those exhibited by drug-loaded PLGA NPs that we generated in a separate study.^37^ QDs were visible as discrete electron-dense domains within NPs (Figure 3B) when imaged using transmission electron microscopy (TEM) without uranyl acetate negative staining.^38^ QD-NP cores contain 14.96 ± 2.5 QDs/NP, with no statistical differences between NP lots (Figure 3C).

**Figure 3:**
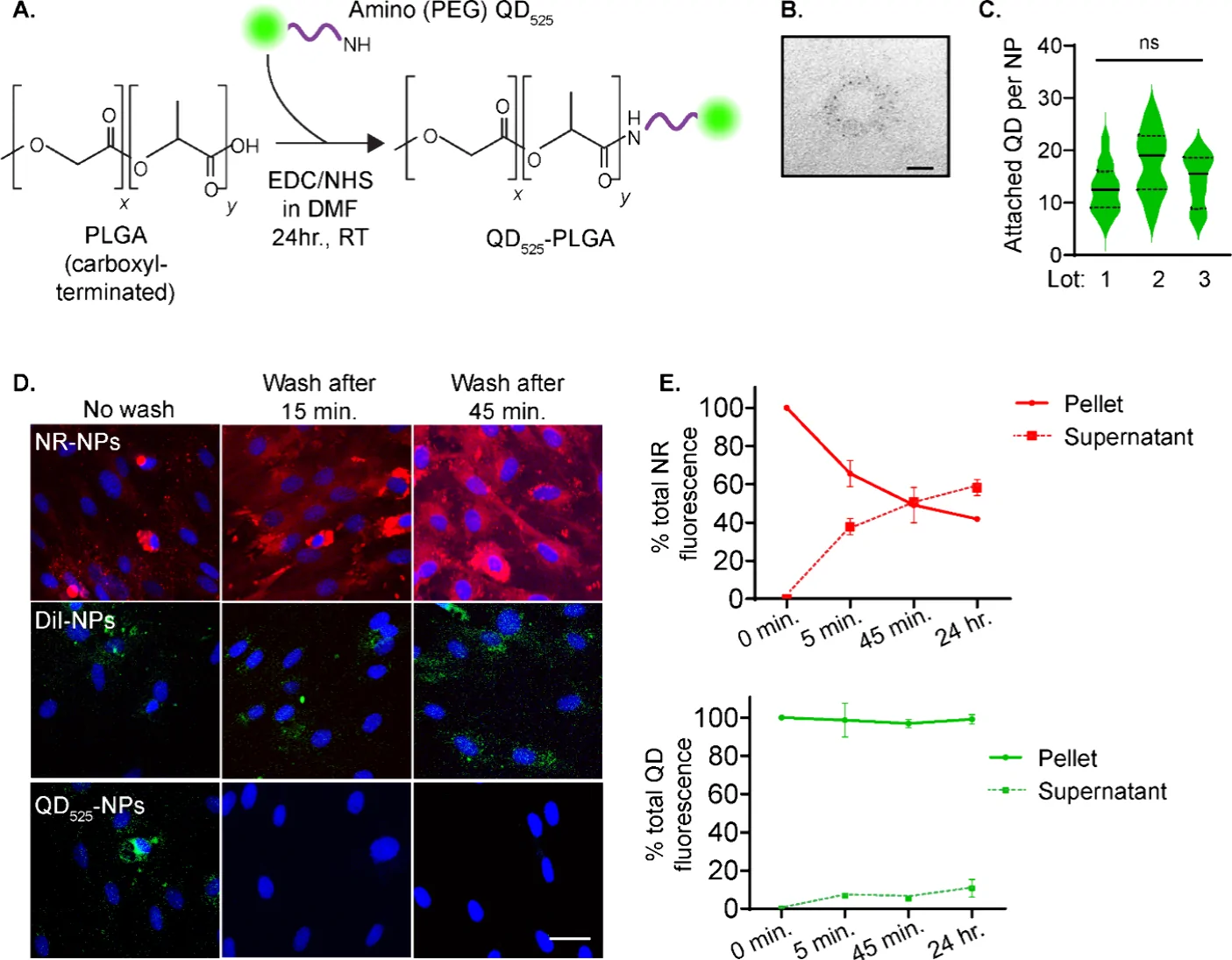
Quantum dot labeling of PLGA polymer. Schematic in (A) depicts covalent attachment of QDs to PLGA via carbodiimide coupling. A representative transmission electron micrograph depicting a QD-conjugated NP core is shown in (B). Attached QDs per NP were quantified (C) over three separate lots of QD-NPs. Scale bar, 20 nm; lines in violin plot indicate median and interquartile range, *n* = 20 NP/lot. (D) Nile Red (NR), 1,1′-dioctadecyl-3,3,3′,3′-tetramethylindocarbocyanine perchlorate (DiI), or QD_525_-labeled NPs were incubated with quiescent PNMECs for 15–45 min before washing and imaging, as indicated. Scale bar, 30 μm. (E) NR-NPs and QD-NPs were suspended in PBS containing 50% FBS for 0–24 h. At indicated intervals, NP were pelleted, and fluorescence in the pellet and supernatant was quantified. Data shown are the mean ± SEM, *n* = three separate tubes per time point.

QD retention in the NP core was assessed in comparison to conventional fluorescent dyes. NPs loaded with Nile red (NR) or 1,1-dioctadecyl-3,3,3′-tetramethylindocarbocyanine perchlorate (DiI), or conjugated with QD_525_, were applied to monolayers of quiescent PNMECs for defined intervals before washing and imaging. NR-loaded NPs exhibited rapid dye leakage into cells at 15 min (Figure 3D, top row), whereas DiI-loaded NPs exhibited visible dye leakage into cells at 45 min leading to diffuse intracellular fluorescence (Figure 3D, middle row). In contrast, QD-labeled NP did not exhibit any dye diffusion into monolayers (Figure 3D, bottom row), demonstrating the superior labeling stability of QDs under physiological conditions. These *in vitro* findings are further supported by our demonstration that NR-NPs in suspension lose over 30% of loaded fluorescence to the supernatant after 5 min of suspension in buffer (Figure 3E, top panel), and this dye transfer persists over a 24h period. QD-NP do not lose any NP-bound fluorescence to the supernatant over 24h when in suspension (Figure 3E, bottom panel).

### Optimization and Characterization of mNPs

To generate BNB targeted NPs, we coated QD_525_-labeled PLGA NP cores with macrophage-derived plasma membrane vesicles by probe sonication using our previously established methods.^35^ To determine the optimal ratio of NP and membrane vesicle components such that neither component was in excess, we assessed the colloidal stability of NPs coated with varying amounts of membrane material. Size (Figure 4A) and PDI (Figure 4B) of NP and membrane vesicle suspensions in 1× PBS were assessed at baseline and after 4 or 7 days of constant agitation. Uncoated QD-NPs (NP only) exhibited significant increases in both size (from 151.83 ± 0.16 nm to 235.06 ± 5.9 nm) and PDI (from 0.04 ± 0.004 to 0.2 ± 0.035) over time, consistent with colloidal instability and NP aggregation. Pure suspensions of membrane vesicles were also unstable, increasing in both size (from 307.5 ± 2.48 nm to 960.7 ± 15.26 nm) and PDI (from 0.37 ± 0.01 to 0.59 ± 0.007). Significant increases in size (140.7 nm ± 0.5 nm to 198.36 ± 16 nm) and PDI (0.06 ± 0.002 to 0.2 ± 0.035) were also observed at the membrane protein:NP weight ratio of 0.05, indicating suboptimal coating and aggregation. In contrast, a membrane protein:NP weight ratio of 0.1 represents the minimal amount of membrane material needed to yield NPs with stable size and PDI from Day 0 through Day 7.

**Figure 4:**
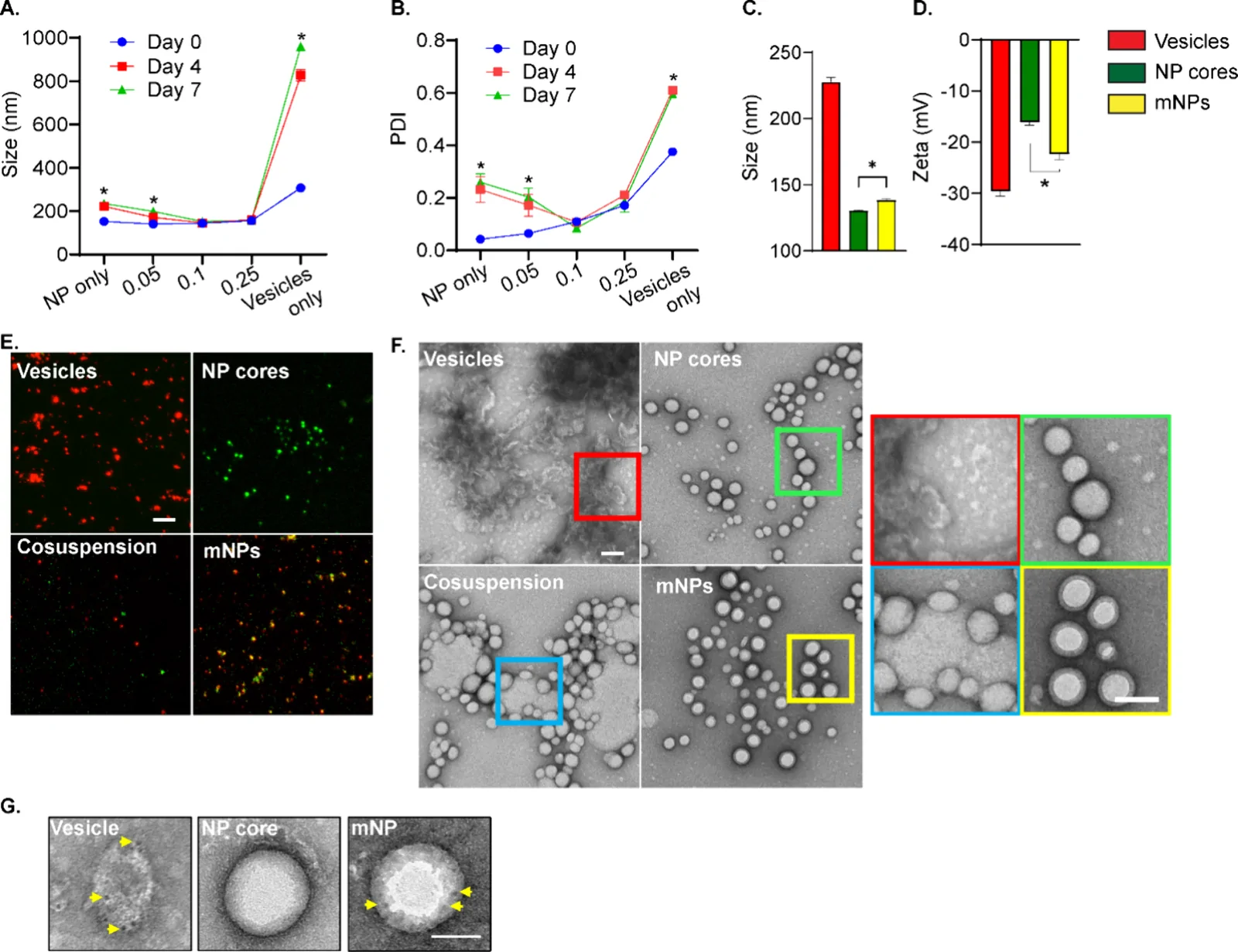
Characterization of macrophage membrane coated NPs. (A–D) Co-suspensions of different indicated ratios of membrane vesicles and QD-labeled PLGA NP cores were sonicated to induce membrane coating and then incubated in 1× PBS under constant agitation. Size (A) and PDI (B) were assessed by dynamic light scattering on Day 0 (after sonicating), Day 4, and Day 7. Data shown are the mean ± SEM, *n* = 3. *p < 0.05 Day 0 versus both Day 4 and Day 7 for the indicated ratios, two-way ANOVA with Tukey’s post hoc test. Size (C) and zeta potential (D) of membrane vesicles, NP core, and coated mNPs were assessed under optimized conditions. Data shown are the mean ± SEM, *n* = 4. *p < 0.05 as indicated, one-way ANOVA with Tukey’s post hoc test. (E–G) Representative confocal (E) and TEM (F, G) images depicting suspensions of DiI-labeled membrane vesicles, QD_525_-NP cores, cosuspensions of the two, or membrane coated NPs (mNP). Arrows in (G) indicate immunogold labeled membrane proteins CD11b and CD18. Scale bars: E, 10 μm; F, 100 nm; G, 50 nm.

Membrane coating onto NPs was confirmed for each experiment using dynamic light scattering, confocal microscopy, and TEM. NPs consistently exhibit a statistically significant shift in size (+8 to 12 nm, Figure 4C) and zeta potential (Figure 4D) upon coating. Representative confocal images (Figure 4E) and electron micrographs (Figure 4F) depict membrane vesicles, NP cores, cosuspensions of the two components, and coated mNPs. Using TEM, globular membrane aggregates with associated NPs are apparent in cosuspensions of NPs and vesicles that were not sonicated to induce NP coating (blue inset, Figure 4F). After sonicating under optimized conditions, we observe an absence of membrane:NP aggregates and a distinct membrane shell around NPs (yellow inset, Figure 4F). Sonication-induced membrane coating is also demonstrated by the colocalization that emerges only after cosuspensions are sonicated (Figure 4E, bottom panels). Finally, immunogold labeling (Figure 4G) depicts the presence of electron dense, CD11b/CD18^+^ puncta (arrows) in membrane vesicles (left panel) and the outer surface of mNPs (right panel). Collectively, these data demonstrate that a membrane protein to NP weight ratio of 0.1 yields stable and reproducible membrane coating of QD-NP cores and retention of key targeting proteins.

### Specificity of mNPs to Inflamed BNB *in Vitro*

Firm adhesion of leukocytes to the inflamed BNB is mediated by specific leukocyte-endothelial cell interactions that can be mimicked *in vitro*.^25,39^ Thus, after establishing the individual NP, immune and primary endothelial cell components and characterizing the optimized mNP formulation, we sought to determine the ability of macrophage plasma membrane coating to enhance specificity of NPs to inflamed BNB *in vitro.* Minimal QD_525_-associated fluorescence was observed when we applied bare NPs to quiescent or TNFα-stimulated PNMEC monolayers (Figure 5A). Similar findings were observed when we applied mNPs to quiescent PNMECs, suggesting that at baseline, the peripheral nerve microvasculature does not express moieties that recruit circulating leukocytes. QD_525_-associated fluorescence was observed on PNMEC monolayers that were stimulated with TNFα and incubated with mNPs (Figure 5A), with an increase (*p* < 0.001) in the corrected total cellular fluorescence (CTCF) compared with all other conditions (Figure 5B). Given our validation that QD-associated fluorescence does not leach into the monolayer (Figure 3C), these findings suggest that macrophage membrane coating promotes direct engagement of NPs with the inflamed BNB.

**Figure 5:**
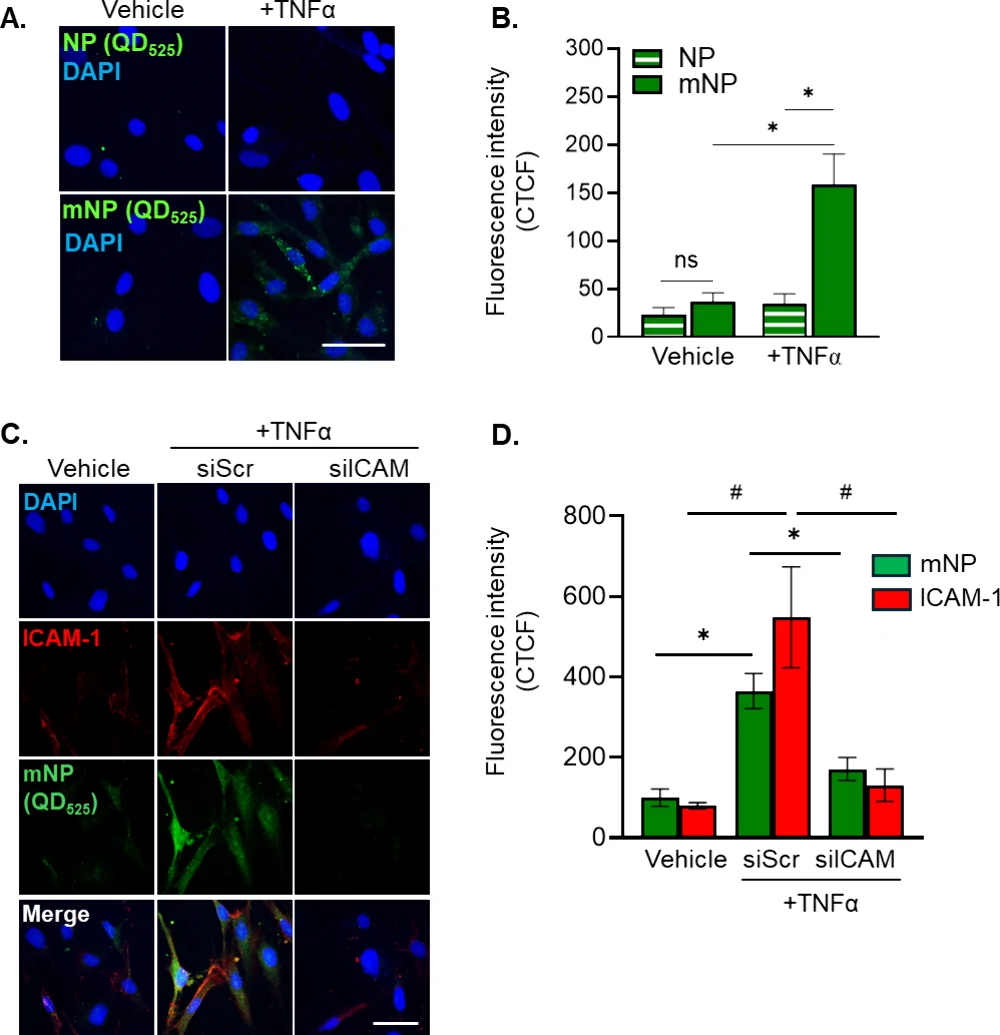
Evaluating mNP specificity to inflamed BNB *in vitro*. PNMEC monolayers treated with vehicle or TNFα (25 ng/mL) were incubated with bare NP or mNP (300 ug/mL) for 2 h before washing and imaging (A). Quantified corrected total cellular fluorescence (CTCF) is shown in (B). In a separate experiment, PNMEC monolayers were transfected with ICAM-specific siRNA or scrambled siRNA control before vehicle or TNFα treatment and mNP incubation. Representative images (C) show ICAM-1 (red) and mNP (green) expression in all conditions as indicated. Quantified CTCF is shown in (D). Scale bars in A and C, 50 μm. Data in B and D are the mean ± SEM, *n* = 4–6. *^,#^, *p* < 0.05 as indicated, one-way ANOVA with Tukey’s post hoc test.

We investigated the involvement of the ICAM-1/LFA-1 signaling axis on mNP adhesion to inflamed PNMECs with siRNA knockdown of endothelial ICAM-1. We first validated knockdown of ICAM-1 protein in TNFα stimulated PNMECs using Western immunoblot (Supplementary Figure S1) and confocal microscopy (Figure 5C). PNMECs transfected with scrambled control siRNA (siScr., 25 nM) and stimulated with TNFα exhibited robust ICAM-1 protein expression (#, *p* < 0.0001) compared with quiescent controls (Figure 5D), while PNMECs transfected with ICAM-1 siRNA (25 nM) exhibited ICAM-1 protein levels that were statistically indistinguishable from quiescent cells (Figure 5C, D). To determine the functional relevance of ICAM-1 knockdown, we assessed mNP specificity to ICAM-1 depleted, inflamed PNMECs. We observed a significant decrease (*, p = 0.0176) in mNP fluorescence associated with ICAM-1-depleted, inflamed PNMECs compared with scrambled siRNA transfected PNMECs (Figure 5D). Together, these results demonstrate increased specificity of mNPs to inflamed BNB *in vitro* through integrin-mediated adhesive mechanisms.

### Fate of mNPs in Organs and Nerves over the Course of EAN

We previously established that EAN-associated BNB permeability over the course of EAN facilitates accumulation of circulating PEGylated NPs within affected peripheral nerves at intermediate and peak stages of disease.^18^ Given that ICAM-1 is expressed by BNB endothelial cells as early as EAN onset, but not constitutively, we hypothesized that leukocyte membrane coating would enhance NP recruitment at early disease stages that are a critical time for administering potential therapies. As shown in Figure 6A, we administered QD_525_-labeled, DiR loaded NPs with or without macrophage plasma membrane coating intravenously to rats at either EAN onset (Day 11) or peak (Day 15) followed by perfusion with saline 24 h. later. Using an optical barcoding strategy,^36^ DiR fluorescence was used to assess and quantify gross biodistribution within organs (Figure 6B– [Fig fig6]E) and nerves (Figure 7A, 7B). QD_525_ labeling was used to delineate precise cellular NP fate with confocal and structured illumination microscopy (Figures 7–8).

**Figure 6:**
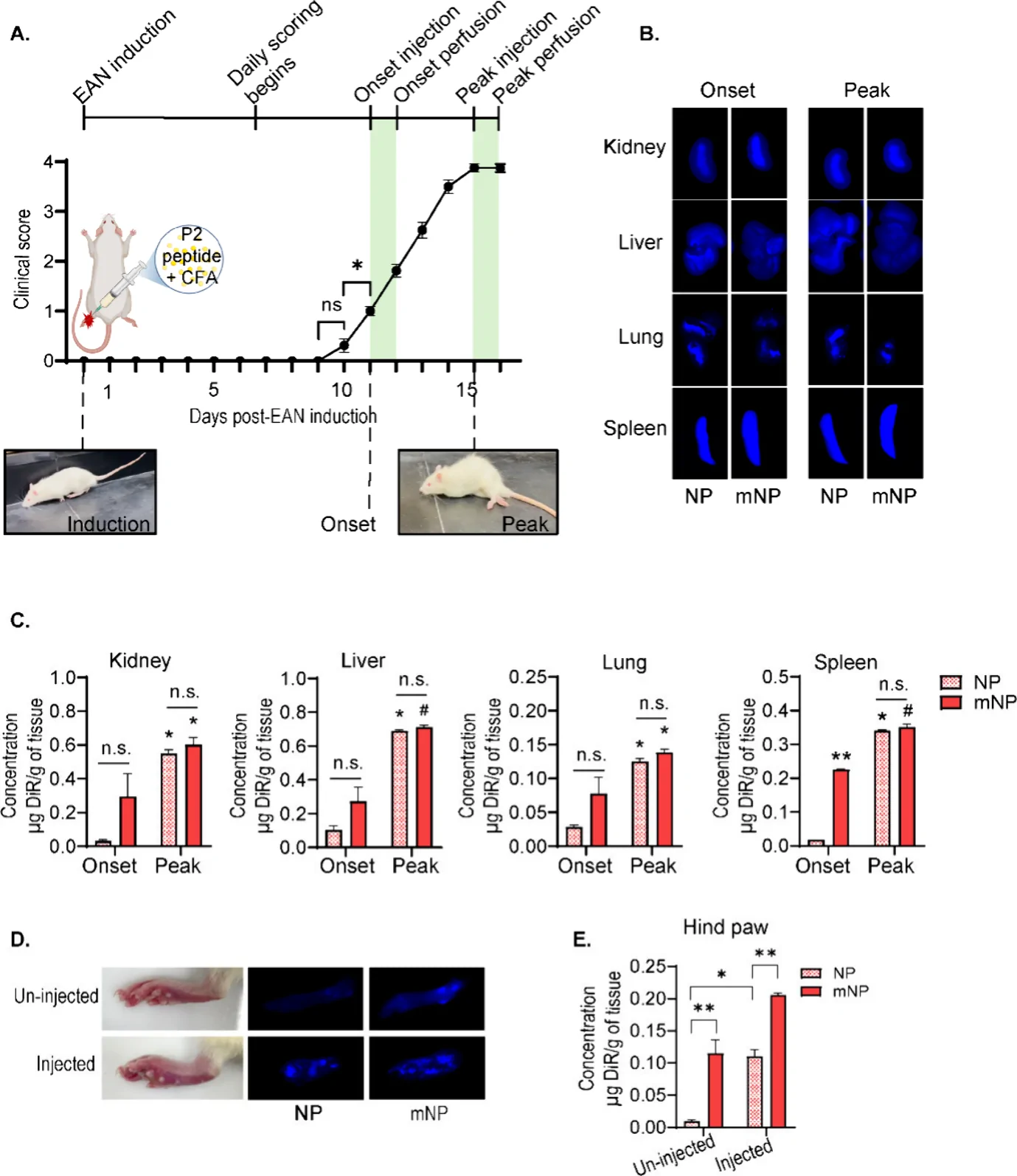
Biodistribution of NP and mNP in rats at EAN onset and peak. (A) Rats received a footpad injection of P2 peptide and complete Freund’s adjuvant for EAN induction on Day 0. At EAN onset (Day 11) or EAN peak (Day 15), rats received a tail vein injection of DiR-loaded, QD-labeled NPs or mNPs. After 24 h circulation, rats were perfused with saline and organs imaged *ex vivo* (B, D). DiR content in kidney, liver, lung, spleen, and hind paw was extracted and quantified (C, E). Data shown in C and E are the mean ± SEM, *n* = 4 rats/group; each organ was analyzed using a two-way ANOVA followed by Tukey’s multiple comparison analysis. In C: *, *p* < 0.05 vs onset NP; **, *p* < 0.001 vs NP; ^#^, *p* < 0.05 vs onset mNP. In E: *, *p* < 0.01 vs uninjected NP and **, *p* < 0.01 vs NP as indicated.

**Figure 7:**
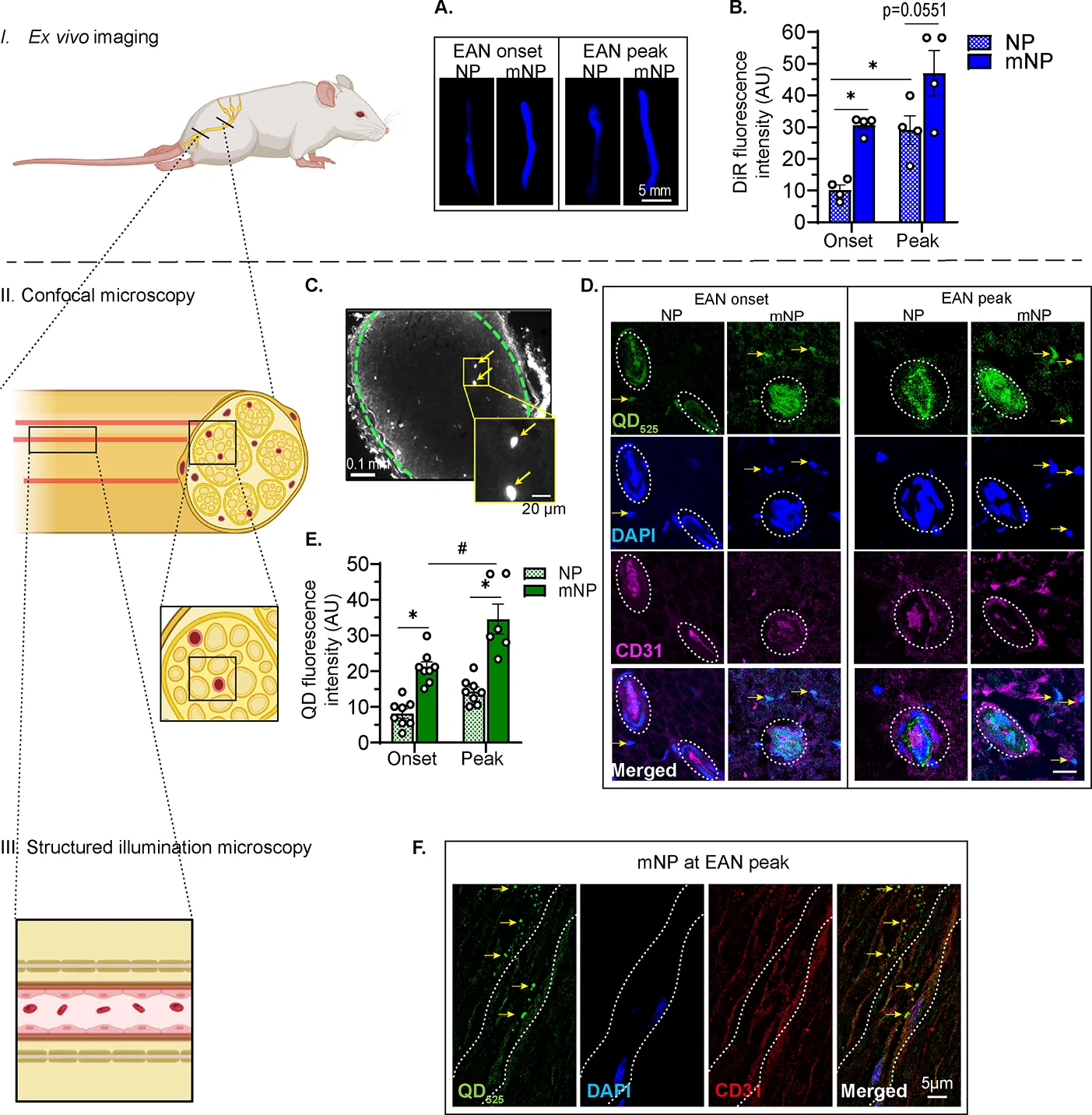
Intraneural fate of mNP. NP entry into nerves was evaluated at three levels of resolution: *ex vivo* in whole sciatic nerves (I: A–B), with confocal imaging in cross sections (II: C–E), and with super-resolution structured illumination microscopy (SIM) in longitudinal sections (III: F). I: DiR fluorescence intensity was used to assess gross distribution across a 2 cm length of nerve (Scale bar in A, 5 mm). Data shown in (B) are the mean ± SEM, *n* = 4 nerves. *p < 0.05, as indicated, two-way ANOVA with Tukey’s multiple comparison. II: Cross section in (C) is from a rat at EAN onset that received an IV infusion of Evan’s blue dye to illustrate the epineurium (green dashed line) and endoneurial vessels (arrows). Scale bar in cross section, 0.1 mm; in inset, 20 μm. Representative confocal images (D) depict CD31^+^ endoneurial vessels and overlaid regions of interest (ROI, dotted lines). Arrows indicate QD-positive cells beyond defined ROIs. Scale bar, 20 μm. Fluorescence intensity in ROI around endoneurial vessels was quantified (E). Data shown are the mean ± SEM, *n* = 6–8 nerves, *p < 0.05 and ^#^ *p* < 0.001, as indicated, two-way ANOVA with Tukey’s multiple comparison. III: Representative SIM images (F) depict QD_525_ puncta (arrows) and CD31^+^ endoneurial vessels in longitudinal sections from a rat that received mNP at EAN peak. Scale bar, 5 μm.

**Figure 8:**
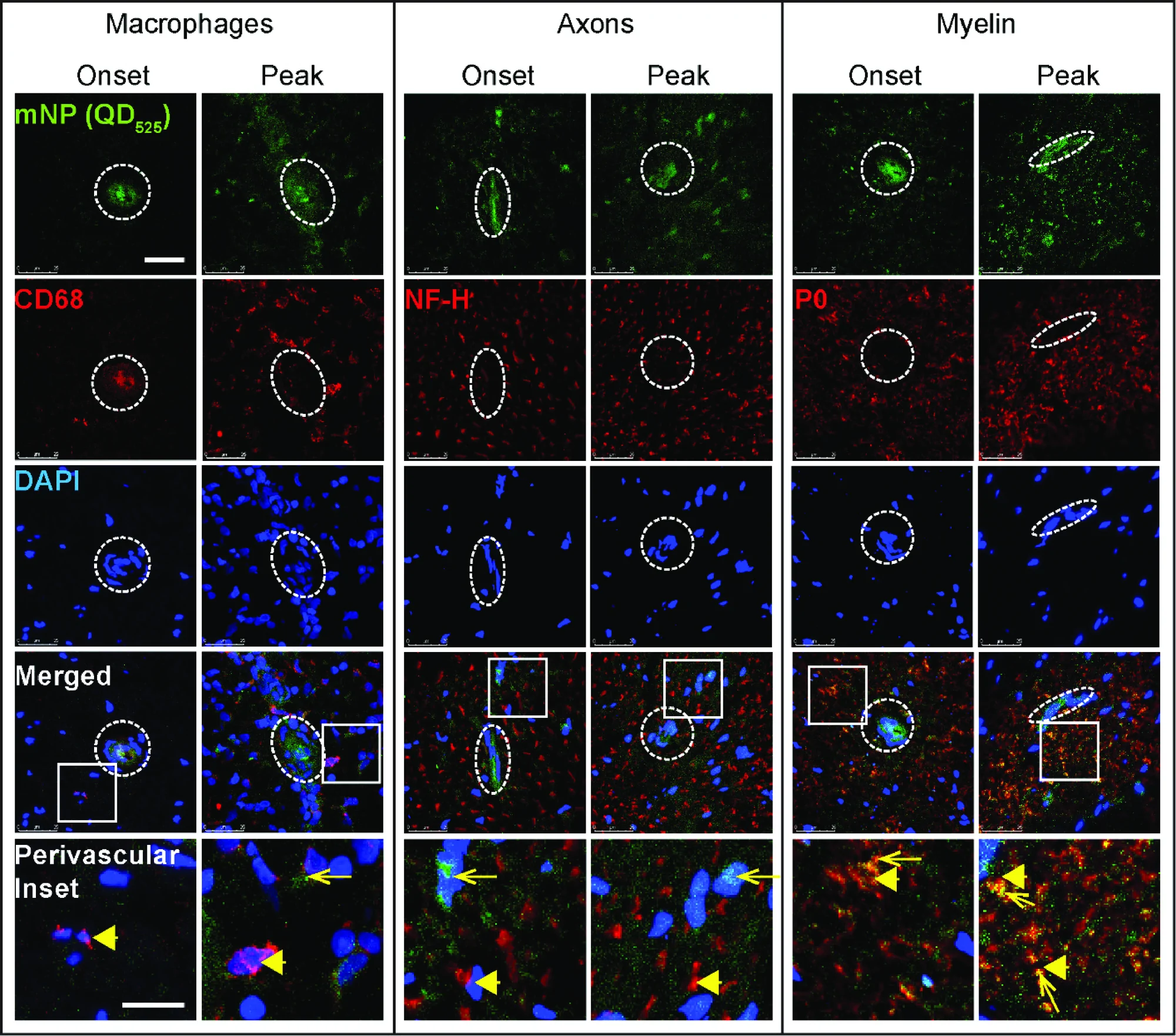
Cellular association of mNP with intraneural targets. Sciatic nerve cross sections were immunolabeled for CD68^+^ macrophages, axons (neurofilament heavy chain, NF-H), or myelin (myelin protein zero, P0), shown in separate panels (red), with mNPs shown in green. Representative confocal images from EAN onset and peak are shown. Dotted lines indicate endoneurial blood vessels, defined by anatomical hallmarks. Solid boxes in merged images indicate perivascular insets, which are shown in the bottom row. Yellow arrowheads, immunolabeled cells; arrows, mNP-positive cells. Scale bar in nerve sections, 25 μm; scale bar in perivascular insets, 10 μm.

Biodistribution of DiR-labeled NPs and mNPs in EAN animals at EAN onset and peak (Figure 6A) was first evaluated through *ex vivo* fluorescence imaging of major organs (Figure 6B). Fluorescence signals observed in the liver, spleen, and kidney for both treatment groups indicate clearance through the reticuloendothelial system. In brain and spinal cord (Supplementary Figure S2), no DiR signals or macrophage infiltration were detected, highlighting the peripheral nerve-specific inflammatory processes occurring during EAN. Quantitative analysis of DiR in organ homogenate (Figure 6C) found that at EAN onset, mNPs exhibited a greater presence in spleen compared with NPs (**p < 0.001, mNP vs NP). This agrees with an earlier finding that macrophages, particularly pro-inflammatory M1 macrophages, are increased in the spleen after EAN onset.^40^ There was no statistical difference in organ accumulation of NPs vs mNPs at EAN peak (n.s., Figure 6C); however, all organs exhibited a statistically significant increase in NP accumulation at EAN peak compared with EAN onset, regardless of NP surface (*, *p* < 0.05 vs onset NP; ^#^, *p* < 0.05 vs onset mNP, Figure 6C). We then assessed NP localization within 10 μm sections of organs using confocal microscopy (Supplementary Figure S3). Both NP and mNP formulations exhibited detectable accumulation within parenchymal regions of the liver and spleen, with relative increases in QD-NP fluorescence at EAN peak compared with EAN onset in those organs. No notable differences in NP localization patterns were observed between the two groups, corroborating the quantitative *ex vivo* findings.

To induce EAN, rats receive a single, unilateral hind footpad injection of P2_53–78_ neuritogenic peptide and complete Freund’s adjuvant on Day 0 (Figure 6A). As autoimmune demyelination develops, rats also develop adjuvant-associated local injection of one hind paw (Figure 6D, left panels). To better understand NP distribution throughout the body as well as inflammation-specific targeting, we assessed NP and mNP distribution in hind paws. Using *ex vivo* imaging, NP accumulation was observed in CFA-injected, but not uninjected paws (Figure 6D, middle panels), and membrane coating increased NP accumulation in both paws (Figure 6D, right panels). We then quantified DiR contained within organ homogenate (Figure 6E). These findings further support the inflammation-specific targeting of mNPs, with local inflammation in adjuvant-injected paws enabling passive recruitment of bare NPs (*p < 0.01, Figure 6E) and demyelination-associated damage to the tibial nerve in both uninjected and injected paws promoting additional recruitment of mNPs (**p < 0.01, Figure 6E).^12^

Our first assessment of NP and mNP accumulation in nerves was via *ex vivo* imaging of freshly harvested whole nerves (see Figure 7, section I). Representative images (Figure 7A) depict DiR fluorescence in a 2 cm length of nerve. Relative fluorescence was quantified, and values plotted for each nerve (n = 4 nerves from 4 rats per group). At EAN onset (*p < 0.05) and peak (*p* = 0.0551), nerves from animals receiving mNPs were brighter than those from animals receiving NPs (Figure 7B). In pilot studies, we found that DiR extracted from nerves was below the level of sensitivity for quantification on our plate reader (data not shown), so we proceeded with localization of QD-NPs using confocal and structured illumination microscopy.

### Intraneural Distribution of mNPs over the Course of EAN

Circulating cells and NPs gain access to nerves by extravasating across the inflamed BNB and entering the endoneurial space. Therefore, to evaluate distribution and localization of QD-NPs, we analyzed sciatic nerve cross sections with confocal microscopy (see Figure 7, section II), focusing on the areas (see inset) surrounding endoneurial blood vessels (yellow arrows in cross section and inset, Figure 7C). We quantified fluorescence in regions of interest around CD31^+^ endoneurial vessels in serial sections. Representative cross sections (Figure 7D) show green QD_525_-associated fluorescence around and within magenta CD31^+^ vessels that are indicated by a white dashed line (1 – 2 vessels per image). At both EAN onset and EAN peak, compared with NPs, mNPs are observed deeper within the endoneurium and are associated with DAPI positive nuclei (indicated by yellow arrows in Figure 7D). This suggests that macrophage membrane coating may allow NPs to engage with nerve-associated cellular targets. Analysis of cohorts (3 vessels/nerve, n = 6–8 nerves per group) demonstrated that macrophage membrane coating significantly increased NP penetration into nerves from endoneurial vessels (Figure 7E) at both EAN onset (*p < 0.001) and EAN peak (*p < 0.0001). Furthermore, penetration of mNPs, but not bare NPs, was greater at EAN peak than at EAN onset (^#^ *p* < 0.001, Figure 7E).

To strengthen our characterization of QD-NP penetration into nerves, we complemented conventional confocal microscopy with super resolution structured illumination microscopy (see Figure 7, section III). In a representative longitudinal section (Figure 7F), QD puncta (yellow arrows) are observed along CD31^+^ endoneurial vessels (dashed lines) with greater spatial resolution. Collectively, these orthogonal approaches depict disease stage and surface modification-dependent accumulation of NPs in affected nerves in EAN.

Finally, to begin to identify the cellular targets of mNPs within the endoneurium, we performed immunohistochemical labeling of CD68^+^ macrophages, neurofilament heavy chain (NF-H), and myelin protein zero (P0) in nerve cross sections collected from rats receiving mNPs at EAN onset or peak (Figure 8). In representative images, dashed lines depict approximate endoneurial vessel locations based on QD_525_ fluorescence (green) and distribution of anatomical hallmarks; these sections were not labeled with a CD31 antibody. Solid boxes in merged images indicate perivascular insets containing both mNPs (green) and cell types of interest (red). Z-stacked confocal images revealed no colocalization of mNPs (arrows) with CD68^+^ macrophages or neurofilament-positive axons (arrowheads, see merged images and insets, Figure 8). In contrast, colocalization of mNPs (arrows) with P0 (arrowheads) was observed at EAN onset and more pronounced at EAN peak (Figure 8). These findings suggest that after recruitment to the BNB and accumulation in the endoneurial space, macrophage membranes may promote association with damaged myelin.

## Discussion

There is a pressing need to develop targeted therapeutics for autoimmune neuropathies. Of note, there are several promising examples of pathological mechanisms and therapeutic targets that have been identified in the EAN model of AIDP/GBS.^6,8–10^ Unfortunately, clinical translation of these findings is limited, given the challenges associated with drug delivery to nerves.^41^ Peripheral nerves are both ubiquitous, with distribution throughout the limbs and trunk, and anatomically restricted by layers of connective tissue and specialized endothelium (the BNB). Current treatment strategies do not leverage disease-specific pathophysiological changes that could enable selective targeting of inflamed peripheral nerves. To fill this gap, we are focused on developing targeted NP delivery systems that can access sites of nerve inflammation. Previous studies have employed an array of leukocyte-derived cell membranes for active NP targeting to inflamed lung,^42^ heart,^43^ or joints.^25^ Here, we leveraged established immune cell-endothelial interactions at the BNB, specifically, integrin-mediated binding to upregulated ICAM-1,^5,26^ to maximize NP delivery to inflamed nerves. Our platform consists of rat macrophage membrane coated, stably labeled polymeric NPs. This design is informed by the significant presence of infiltrating myeloid cells over the course of EAN,^7^ the integrin-rich plasma membrane profile of macrophage cell lines,^44^ and the upregulation of ICAM-1 by BNB endothelial cells at EAN onset that we previously identified.^18^

By using polymer with covalently conjugated quantum dots (Figure 3),^36^ we were able to precisely track NP fate within the microvasculature of affected nerves and identify mNP interactions with endoneurial cellular targets over the course of EAN (Figure 8). Despite robust accumulation within the endoneurial compartment, mNPs did not colocalize with CD68^+^ macrophages or neurofilament-expressing axons, suggesting that they are not internalized by infiltrating immune cells or recruited toward axons. The lack of direct axonal association may be beneficial, given the vulnerability of axons during inflammatory demyelination.^45^ In contrast, mNPs exhibited colocalization with myelin associated protein P0 over the course of disease, suggesting that macrophage membrane coating may impart affinity for damaged or exposed myelin components. It is established that inflamed Schwann cells express ICAM-1, VCAM, and other inflammation-associated adhesion molecules and cytokines,^46^ and Schwann cells can actively recruit immune cells.^47^ Such specificity has important implications for payload selection, as mNPs may be particularly well suited for the delivery of agents aimed at modulating demyelination, promoting remyelination, or stabilizing myelin structure.

An extensive body of work with the rat EAN model has identified several classes of therapeutic agents that attenuate disease by engaging with targets within nerves when administered systemically. These compounds, including sphingosine-1-phosphate receptor modulators,^8,9^ statins,^6^ sodium channel blockers,^12^ and antimitotic compounds,^10^ represent potential payloads for this delivery system. While translation of preclinical findings is limited by systemic toxicity, insufficient drug concentrations within nerves, and off-target effects, this mNP-based delivery strategy provides an opportunity to locally concentrate payloads within inflamed peripheral nerves at early disease stages (Figure 7). By intervening upstream of extensive myelin loss, this approach may not only enhance the efficacy of candidate therapeutics but also reduce the overall duration and dose of drug exposure required. Enhanced exposure to nerves using this modality is further supported by the diffuse presence of loaded DiR in nerves (Figure 7A). While attached QDs enabled fate-tracking with high resolution,^36^ we (see DiI-NPs in Figure 3) and others have shown that loaded carbocyanine dyes are not retained in NP cores, in practice serving as a preliminary payload mimic *in vivo*.^48^

In addition to the dual-labeled NP core, the rat macrophage membrane surface represents a refinement to existing cell membrane functionalized delivery systems. *In vivo* studies of NP biodistribution, pharmacokinetics, and efficacy are disproportionately performed in mouse models.^49^ The species from which membrane vesicles are derived plays a role in immunogenicity.^50^ Given that the Lewis rat EAN model is robust and well-characterized for assessing BNB associated pathology,^51^ it was imperative that we identify a rat-specific source of leukocyte plasma membranes. Others have investigated rat cell membranes to coat NPs, including autologously derived primary neutrophil plasma membranes^52^ and cancer cell membranes.^53^ In one example, rats received mouse macrophage-coated NPs; however, in this example, toxicity of the nanoformulation was observed.^54^ NR8383 macrophages remain the only commercially available rat-derived myeloid cell line. Here, we validated its use as an intrinsically active, continuous source of integrin-enriched plasma membranes that adheres to TNFα stimulated, but not quiescent, rat peripheral nerve microvascular endothelial cells (Figure 1).

After identifying a source of plasma membranes, we incorporated the use of nitrogen cavitation to rupture cells (Figure 2). Nitrogen cavitation offers advantages over mechanical homogenization by enabling rapid, reproducible cell lysis while preserving membrane protein integrity.^55^ This is especially relevant for immune membrane-coated nanocarriers, where retention of functional adhesion molecules is critical for biomimetic targeting.^56^ Nitrogen cavitation is also inherently scalable and amenable to batch processing, supporting its translational relevance compared with low-throughput mechanical methods.^57^ Subcellular fractions were isolated from the cavitate using differential centrifugation. While density gradient centrifugation may further reduce the observed presence of histone proteins in the plasma membrane fraction,^58^ it also tends to reduce the amount of plasma membrane material recovered, due to the homogeneity in plasma membrane density and material loss at the sucrose interface. We have not identified any *in vitro* or *in vivo* functional implications of this histone contamination. During platform optimization, we further observed that lyophilization disrupted retention of membrane-associated G protein coupled chemokine receptors (data not shown), highlighting the importance of processing conditions in maintaining functional membrane coatings.

The robustness and consistency of this platform both *in vitro* and *in vivo* support its broader application in other preclinical disease applications that are ideally modeled in rats, including models of stroke,^59^ traumatic brain injury (TBI),^60^ and spinal cord injury.^61^ Like AIDP/GBS, these nervous system injuries and pathologies feature macrophage accumulation and immune cell trafficking across activated blood-nervous system barriers.^62–64^ In addition to these examples, our findings are also logically translatable to chronic inflammatory demyelinating polyneuropathy (CIDP).^4^ The ability of mNPs to exploit conserved inflammatory homing mechanisms and the chronic or relapsing-remitting nature of disease suggest a rational path toward targeted drug delivery in CIDP. Similarly to AIDP, selective enrichment of mNPs at diseased nerves may improve therapeutic efficacy while minimizing off-target exposure.

We previously found that PEGylated NPs can passively accumulate in peripheral nerves at peak disease in EAN.^18^ While this finding uniquely demonstrated that the pathology associated with inflammatory neuropathy provides an opportunity for BNB crossing and nerve delivery, the ideal window for delivering a therapeutic is earlier.^21^ Our findings show that macrophage membrane coating significantly enhances NP accumulation in sciatic nerves as early as disease onset (Figure 7). GBS remains the leading cause of acute flaccid paralysis worldwide, with neurological deficits typically progressing rapidly and reaching maximum severity around 4 weeks after onset. Diagnosis is supported by clinical features, biomarkers, and electrophysiological abnormalities. These confirmatory tests often evolve over the first 1–2 weeks following symptom onset, leaving a window after diagnosis and before disease peak when IV administration of nanomedicine may be beneficial. Current standard-of-care approaches, including IVIg and plasma exchange, are not effective for all patients. Many experience severe complications such as respiratory failure, contributing to significant morbidity and mortality.^65^ Even more prevalent are persistent sensory deficits and chronic neuropathic pain despite receiving therapeutic interventions. Together, these limitations underscore the need for strategies that directly target inflamed peripheral nerves earlier in the disease course, with the goal of intervening before peak severity to preserve myelination and disrupt the downstream inflammatory cascades that are likely to drive long-term neurological deficits, even when definitive diagnosis is still evolving.^3^

As a practical consideration for clinical translation, our *in vitro* stability assay (Supplementary Figure S4) showed that IgG forms aggregates over 7 days, and similar aggregation occurs when IgG is combined with mNPs. Because IVIg is routinely administered in GBS without complications, this observation may not represent a clinical safety concern, but it suggests that future studies may consider dosing strategies to minimize direct interaction between administered IgG and NP formulations.

An additional practical consideration is highlighted by the passive and active accumulation of NPs that we observed in hind paws (Figure 6D and 6 **E**). The local injection of CFA used to induce EAN results in localized inflammation of one hind paw, and our data suggest that this results in recruitment of NP and mNP. Uninjected paws exhibit accumulation of mNP only, likely due to the involvement of tibial nerves that innervate the plantar muscles of the hind paws.^12^ While this finding does not directly bear clinical relevance, given that the local footpad injection is specific to EAN, it does suggest that mNPs may accumulate in additional sites if GBS patients present with preexisting conditions, such as diabetes, heart failure, hyperlipidemia, or chronic systemic inflammation.

Beyond serving as drug carriers, mNPs may also confer intrinsic therapeutic benefit. Prior studies have demonstrated that membrane coated “nanosponge” platforms can adsorb and neutralize circulating pro-inflammatory cytokines, thereby dampening systemic and local inflammation.^66^ Notably, even drug-free polymeric nanoparticles have been reported to attenuate inflammatory responses in preclinical models of AIDP/GBS,^67^ supporting previous findings that polymeric nanomaterials can beneficially modulate immune activity.^68^ Together, these observations raise the possibility that mNPs may provide dual benefit through both targeted delivery and immunomodulation. Nevertheless, immune membrane-based delivery systems pose unique challenges, including scalability of membrane isolation, batch-to-batch variability, and the potential for immune recognition depending on host–donor compatibility. Future optimization may involve standardized membrane sourcing, synthetic or hybrid membrane approaches, or autologous immune cell derivation to mitigate immunogenic risk. Addressing these challenges will be essential for advancing macrophage-mimetic nanocarriers toward clinical translation while preserving their disease-targeting advantages.

## Conclusions

We have developed a nanoparticle (NP) formulation consisting of a rat macrophage plasma membrane coating and a quantum dot labeled polymeric core. Plasma membranes are derived from an intrinsically active and commercially available cell line, and stable labeling of the NP core enables fate tracking within the microvasculature of inflamed peripheral nerves. In a rat EAN model, membrane-associated integrins promoted endoneurial accumulation of NPs and recruitment to Schwann cells within affected nerves. Overall, our findings demonstrate that inflammation-induced changes in the nerve microenvironment can provide opportunities for active targeting of circulating NPs, even before BNB permeability is maximal. Future studies will investigate the functional and therapeutic implications of these findings for delivery of small molecule payloads to nerves, as well as extrapolation of these findings to other disorders of the central and peripheral nervous systems that feature vascular inflammation.

## Methods

### Cell Culture

#### Peripheral Nerve Microvascular Endothelial Cells (PNMECs)

Primary PNMEC cultures were established by freshly dissecting sciatic nerves from naïve Lewis rats and proceeding as we have previously described.^31^ Fibroblasts were selectively lysed by incubating cultures in thy1.1 antibody-containing media collected from TIB-103 hybridoma cells (ATCC) supplemented with 5% rabbit complement (Sigma-Aldrich). PNMECs were maintained in rat tail collagen type I (Gibco) coated flasks, and experimental use was restricted to passages 5–7. In some experiments, PNMECs were transfected with siRNA directed against ICAM-1 (ThermoFisher Scientific, sequence - NM_012967.1, siRNA ID s129863) using our previously published methods.^69^

#### Rat Alveolar Macrophage Cell Line

Macrophages (NR8383, ATCC) were cultured in Ham’s F12K medium (Gibco) with 15% heat-inactivated fetal bovine serum (FBS), 100 unit/ml penicillin, 100 μg/mL streptomycin, and 5.6 μg/mL amphotericin B (Gibco).

### Plasma Membrane Vesicle Isolation and Preparation

Rat macrophages (2.0 × 10^8^) were gently ruptured with nitrogen cavitation (400 p.s.i). Subcellular fractions were collected by stepwise differential centrifugation, with the final 100,000*g* pellet containing plasma membranes. Subcellular fractions were washed, resuspended in normal saline solution (0.9% w/v sodium chloride) with protease inhibitors, and lyophilized for storage at −80 °C until use. Protein profiles of membrane fractions were characterized by Western immunoblot as described below. Lyophilized plasma membrane material was reconstituted in chilled, deionized H_2_O, vortexed vigorously, and centrifuged at 16,000*g* (4 °C, 30 min). The pellet was resuspended in deionized H_2_O and bath sonicated at 50% amplitude, 4 °C, for 5 min (Fisherbrand Model 505 Sonic Dismembrator with cup horn attachment and recirculating chiller (Qsonica LLC)). The sonicated suspension was pelleted and reconstituted as above, and the process was repeated. Vesicle size, polydispersity, and protein retention were assessed after reconstitution with dynamic light scattering and TEM as described further below, and the protein concentration of the processed suspension was quantified using Pierce Rapid Gold BCA Protein Assay Kit (ThermoFisher Scientific).

### NP Preparation

Quantum dot (QD) conjugated NPs were synthesized using methods established by the Sirianni group.^36^ QDs (Qdot_525_ ITK Amino (PEG), ThermoFisher Scientific) consisted of a Cd/Se core and a ZnS semiconductor shell, functionalized by amine-derivatized PEG. First, QDs were covalently coupled to carboxyl-terminated poly­(lactic-*co*-glycolic) acid (PLGA) polymer (50:50, Durect Corp. (Cupertino, CA). Briefly, PLGA-COOH (15 mg, 375 nmoles) was dissolved in dimethylformamide (1.5 mL), and 1-ethyl-3-(3-(dimethylamino)­propyl) carbodiimide (EDC, 6.2 mg), *N*-hydroxysuccinimide (NHS, 4.5 mg) and QDs (120 μL, 0.96 nmoles) were added and stirred for 24h. The polymer was washed with deionized H_2_O and collected by centrifugation at 8000*g* (4 °C, 30 min). NPs were fabricated by mixing QD-PLGA (5 mg) and ester terminated parent PLGA (45 mg). For *in vivo* studies, 0.1 mg 1,1’-dioctadecyl-3,3,3′,3′-tetramethyl-indotricarbocyanine iodide (DiR) was added to the organic phase during NP synthesis. Rat macrophage plasma membrane vesicles were coated on to QD-NP cores at a ratio of 0.1:1.0 w/w (protein to polymer) using bath sonication (60% amplitude, 4 °C, 5 min) as we have previously described.^35^

### NP Characterization

Size and zeta potential of NPs and membrane vesicles were assessed with dynamic light scattering and electrophoretic mobility, respectively, using a Zetasizer Nano ZS90 (Malvern Panalytical). Bare or membrane coated NPs (0.1 mg/mL) were suspended in deionized H_2_O and transferred to a disposable polystyrene cuvette or a disposable capillary cell. NPs were equilibrated to 25 °C for 3 min (for DLS) or 1 min (for zeta potential) in the cuvette and measured at 90° angle. Each sample was measured 4 times. NP coating and QD loading were assessed with transmission electron microscopy (TEM). Carbon-coated 200 mesh copper grids (Electron Microscopy Sciences, Morgantown, PA) were floated on top of 50 μL drops of sample suspensions for 30 min at room temperature. Samples consisted of vesicles, NP cores, or cosuspensions that were sonicated or not. The grids were washed thrice in deionized H_2_O then negatively stained by floating the grid on a 50 μL drop of filtered 2% uranyl acetate (UA, 5 min, room temperature). The sample was allowed to dry (12 h) prior to imaging with a Philips CM 120 transmission electron microscope (TSS Microscopy, Beaverton, OR). To determine the distribution of QDs within NPs, the samples were prepared as described but with the UA negative stain omitted.

To validate retention of targeting ligands by mNPs, we used immunogold labeling following a published method with minor modifications.^70^ Carbon-coated 200 mesh nickel grids were floated on top of 50 μL drops of sample suspensions (30 min, room temperature). Grids were washed in deionized H_2_O, and incubated (30 min, room temperature) with PBS containing 5% BSA, 0.1% cold water fish skin gelatin, and 5% normal donkey serum (Electron Microscopy Sciences). Next, the grids were washed with 0.2% acetylated BSA (BSA-c, Electron Microscopy Sciences) then incubated with PBS-0.2% BSA-c containing 5% BSA, 0.1% cold water fish skin gelatin, 5% normal donkey serum, and 1:200 dilutions of polyclonal rabbit anti-CD18 (ThermoFisher Scientific) and monoclonal rabbit anti-CD11b (clone EPR19373, Abcam) primary antibodies (18 h, 4 °C). Washed grids were incubated in PBS-BSA-c containing donkey antirabbit secondary antibody conjugated to a 4 nm gold particle (1:200, Jackson ImmunoResearch Laboratories, West Grove, PA) for 2 h. at room temperature. Grids were washed with PBS-0.2% BSA-c, incubated with PBS containing 1% glutaraldehyde (15 min, room temperature), washed with deionized H_2_O, and negatively stained with 2% vanadium. Samples were allowed to dry for 12 h. prior to TEM imaging as described above.

### Stability Assays

Colloidal stability was assessed by measuring size and polydispersity index (PDI) of NPs suspended in 1x phosphate buffered saline (PBS) under constant agitation on Days 1, 4, and 7 as we have previously described.^35^ Dye retention was quantified for Nile red (NR) and QD_525_-labeled NPs. Briefly, labeled NPs were reconstituted in PBS containing 50% FBS and incubated for 0 min, 5 min, 45 min, or 24 h. At the designated time, tubes were centrifuged at 15,300 g for 10 min. Fluorescence in collected supernatant and resuspended pellets was measured in triplicate with a plate reader. Total signal at each time point was combined, and the percentage of the total signal was calculated.

### Western Blot

Protein content of subcellular fractions or PNMEC lysates was assessed using a Pierce BCA Protein Assay Kit (ThermoFisher Scientific) according to manufacturer’s protocol. Lysates containing 25 μg protein, Laemmli 4x sample buffer and 50 mM dithiothreitol (DTT) were heated to 95 °C for 5 min, loaded into a 4–20% polyacrylamide gel (Bio-Rad), and resolved by sodium dodecyl sulfate polyacrylamide gel electrophoresis. This was immediately followed by semidry transfer onto nitrocellulose membranes (ThermoFisher Scientific). Blots were blocked (37 °C, 30 min) with buffer containing 5% (w/v) nonfat dry milk in tris-buffered saline with 0.1% (v/v) Tween 20, probed with the indicated primary and corresponding secondary antibodies, and detected using SuperSignal West Pico PLUS Chemiluminescent Substrate (ThermoFisher Scientific). Primary antibodies included rabbit polyclonal anti-CD45 and anti-CD18 (1:500 and 1:1000 dilutions respectively, ThermoFisher Scientific); rabbit monoclonal anti-AIF [D39D2], anti-Histone H3 [D1H2] (1:500 and 1:2000 dilutions respectively, Cell Signaling Technology), and anti-CD11b [EPR19373] (1:1000 dilution, Abcam); and mouse monoclonal anti-GAPDH [1D4] (1:1000 dilution, Novus Biologicals). Secondary antibodies included peroxidase conjugated goat antimouse IgG (H+L, 1:5000 dilution, ThermoFisher Scientific) and goat antirabbit IgG (H+L, 1:2500 dilution, Jackson ImmunoResearch Laboratories). All antibody dilutions were prepared in the blocking buffer described above.

### Adhesion Assays

Primary PNMEC were seeded (2 × 10^4^ cells/cm^2^) in 6-well plates (whole cell adhesion) or 24-well plates (NP adhesion) containing collagen coated glass coverslips (1.5 H thickness; Thorlabs). Confluent monolayers were stimulated with TNFα (25 ng/mL, 24 h Sigma-Aldrich;). Rat macrophages (1 × 10^6^ cells/ml), NP cores or mNPs (300 μg/mL NP for each condition) were added in complete culture media to quiescent or inflamed monolayers for 30 min to 2 h at 37 °C. Nonadhering cells or NPs were removed by gently washing monolayers with 1x PBS. Washed monolayers were fixed with 4% (w/v) paraformaldehyde (PFA), and coverslips were mounted onto slides with Prolong Diamond antifade mountant containing DAPI (ThermoFisher Scientific). Adherent macrophages were counted on a brightfield microscope. Adherent NPs were imaged using confocal microscopy. Images were captured at laser settings of 405, 488, and 561 nm (PMT: 408 – 477 nm, 490 – 555 nm, and 564 – 671 nm respectively) and laser intensity (17.07%, 21.33%, 27.74% respectively), from 4 to 5 regions per coverslip and 3–5 coverslips/group and fluorescence intensity was quantified using ImageJ software.

### EAN Induction

All animal experiments were performed following the protocols evaluated and approved by the Edward Hines, Jr., VA Hospital Institutional Animal Care and Use Committee (Ethics Approval Number: H24–005). Adolescent male Lewis rats (Envigo) were housed in pairs, allowed free access to standard rat chow and water, and maintained on a 10*h*/14h light/dark cycle. EAN was induced in anesthetized rats (ketamine (100 mg/kg)-xylazine (5 mg/kg)) as we have previously described.^32^ Briefly, rats received one hind footpad injection containing 100 μL of a freshly prepared emulsion (1:1 v/v) of 100 μg purified P2 myelin neuritogenic peptide (residues 53–78, Peptide Synthesis Core Facility of the Center for Regenerative Nanomedicine at Northwestern University) suspended in sterile saline and complete Freund’s adjuvant (5 mg/mL heat-inactivated *Mycobacterium tuberculosis*, strain H37RA (ATCC)). Weights and clinical scores were assessed daily beginning on Day 7 postinduction using a standardized scale consisting of: 1 (loss of tail tone), 2 (disrupted gait), 3 (mild hemiparesis), 4 (severe paraparesis), or 5 (paraplegia), and including increments of 0.5, as we have previously described.^32^

### NP Injection, Euthanasia, and Tissue Collection

At EAN onset (Day 11) or EAN peak (Day 15), isoflurane-anesthetized rats received a single, 1 mL tail vein injection of saline containing 20 mg of NPs or mNPs. NPs were allowed to circulate for 24 h. before perfusion with normal saline and euthanasia. Organs (lung, spleen, liver, kidney, brain, spinal cord) and both sciatic nerves and hind paws were collected. One nerve, kidney, spleen, lung, liver, brain, spinal cord, and hind paws were imaged *ex vivo* using an Azure Biosystems 600 imager (Azure Biosystems, Dublin, CA). The remaining tissue was immediately fixed in 4% (w/v) PFA (room temperature, 4 h.) followed by incubation in 30% (w/v) sucrose prepared in PBS (4 °C, overnight). The next day, tissues were incubated in a 50:50 mixture of optimal cutting temperature (OCT) medium and 30% sucrose (w/v) in PBS for 2 h. followed by embedding in pure OCT and snap freezing in liquid nitrogen. OCT-embedded tissue was stored at −80 °C until further analysis.

### Biodistribution Analysis

Immediately after *ex vivo* imaging, nerve and organs were snap frozen and stored at −80 °C until use. Organs were thawed on ice and a portion cut, weighed, and minced with a razor blade. Deionized H_2_O was added for a final concentration of 10% w/v. Tissue was disrupted using a bead homogenizer for 10 min at high speed followed by probe sonication on ice. Homogenate (75 μL) was added to a black-walled 96 well plate and 15 μL of DMSO was added. Fluorescence intensity was read (Ex/Em 725/760) on a SpectraMax i3 plate reader (Molecular devices, San Jose, CA). Control curves were generated for each organ by spiking control organ homogenate with known amounts of DiR and converting arbitrary values to concentrations (μg DiR/g of tissue).

### Immunohistochemistry and Imaging

#### Localization of QD-NPs and Endoneurial Vasculature

Serial 10 μm cross or longitudinal sections were prepared from nerves and organs using a Leica CM 1850 Cryostat and mounted onto Superfrost Plus microscope slides. Sections were washed and blocked (30 min, room temperature) in PBS containing 5% (v/v) normal goat serum and 0.10% (v/v) Triton X-100. Nerve sections were incubated with mouse anti-CD31 primary antibody (1:100 dilution in blocking buffer) followed by Alexa Fluor 594 antimouse secondary antibody (1:1000 dilution in blocking buffer) and coverslipped as described above. NP accumulation around endoneurial vessels in cross sections was quantified with confocal microscopy at 63× magnification at a laser setting of 488 nm (photomultiplier tube = 490–530 nm). Z-stacked images were converted to one image using maximum projection on Leica Application Suite X. Standardized regions of interest (ROI) around CD31^+^ endoneurial blood vessels were selected (up to 10 nerve sections per animal, 4 animals per cohort) and fluorescence intensity was quantified using ImageJ software. Extravasation of mNP puncta was assessed in longitudinal sections using a Lattice SIM 5 microscope (Carl Zeiss Microscopy, Jena, Germany). Raw images were subjected to dual iterative SIM (SIM2) processing with Zen 3.10 software (Carl Zeiss Microscopy, Jena, Germany) with the following parameters: results sampling = 2, processing sampling = 2, sharpness setting = weak, order combination = Wiener filter, regularization weight = 0, regularization = none, iterations = 3, filter = no filter, fitting = fast fit, histogram = scale to min/max.

#### Identification of mNP Cellular Targets

Serial cross sections were prepared from nerves as described above. Slides were incubated with rabbit monoclonal antimyelin protein zero (P0) [EPR20383], rabbit monoclonal antineurofilament heavy polypeptide (NF-H) [EPR20020], or mouse monoclonal anti-CD68 primary antibody [ED-1] (all diluted 1:100 in blocking buffer and obtained from Abcam), followed by corresponding Alexa Fluor 594 conjugated secondary antibody and imaging with confocal microscopy as described above. Endoneurial vessels were identified by the presence of QD-NP foci, clustering of DAPI-stained nuclei, and absence of axonal or glial markers. Peri-vascular inset ROIs were assessed for colocalization of QD-NP and cell types of interest.

### Statistics

All statistical analyses were conducted using GraphPad Prism 10.2.2 (397). Data are expressed as the mean ± SEM of N observations, and *p* < 0.05 was considered statistically significant, denoted with * or #. Nonsignificant observations were denoted as n.s. Statistical significance across experimental groups (*in vitro* adhesion assay, mNP characterization, *in vivo* intraneural localization) was conducted using one-way ANOVA comparison followed by Tukey’s posthoc test. Stability and biodistribution data were analyzed using two-way ANOVA comparison followed by Tukey’s posthoc test.

## Supplementary Material



## Notes

Additional figures, including (S1) Western blot validation of siRNA knockdown, (S2) CNS biodistribution, (S3) QD-NP organ distribution, and (S4) stability assay with IVIg (PDF)
