# Supplementary material for: Macrophage Plasma Membrane Coating Enhances Nerve Delivery of Stably Labeled Polymeric Nanoparticles
Source: ACS Nano Med. 2026 Oct 7;1(10):2730–44. doi: 10.1021/acsnanomed.6c00218 (PMC13761686; doi:10.1021/acsnanomed.6c00218)
Supplement: Supplementary file 1 [file nm6c00218_si_001.pdf]

Supporting information

Macrophage Plasma Membrane Coating Enhances Nerve Delivery of Stably Labeled Polymeric Nanoparticles

Chanpreet Kaur, [1,2] Maleen H. Cabe, [1,2] Charles J. Kasella,[1] Natalie Velazquez,[1] and Kelly A. Langert* [1,2]

Department of Molecular Pharmacology and Neuroscience

Loyola University Chicago, Stritch School of Medicine, Maywood, IL 60153 (U.S.A.) [1]

Research Service

Edward Hines Jr. VA Hospital, Hines, IL 60141 (U.S.A.) [2]

Corresponding author:
Kelly A. Langert, Ph.D.
Dept of Molecular Pharmacology & Neuroscience
Loyola University Chicago
2160 S. First Avenue
Bldg 115, Room 416
Maywood, IL 60153 (U.S.A.)

tel: (464) 220-9598
email: klangert@luc.edu

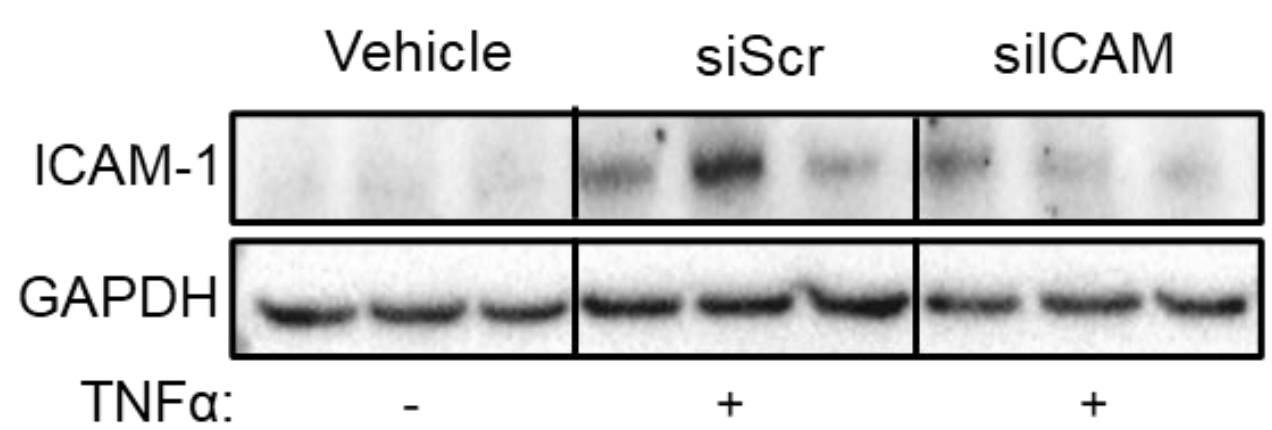

Figure S1. Validation of ICAM-1 siRNA knockdown. PNMEC monolayers were transfected with siRNA control or siICAM (25 nm of each). After 48 hrs, PNMECs were treated with vehicle or TNFα (25 ng/ml), and cell lysates were collected for assessment of ICAM-1 protein content via Western immunoblot.

A. **B.**

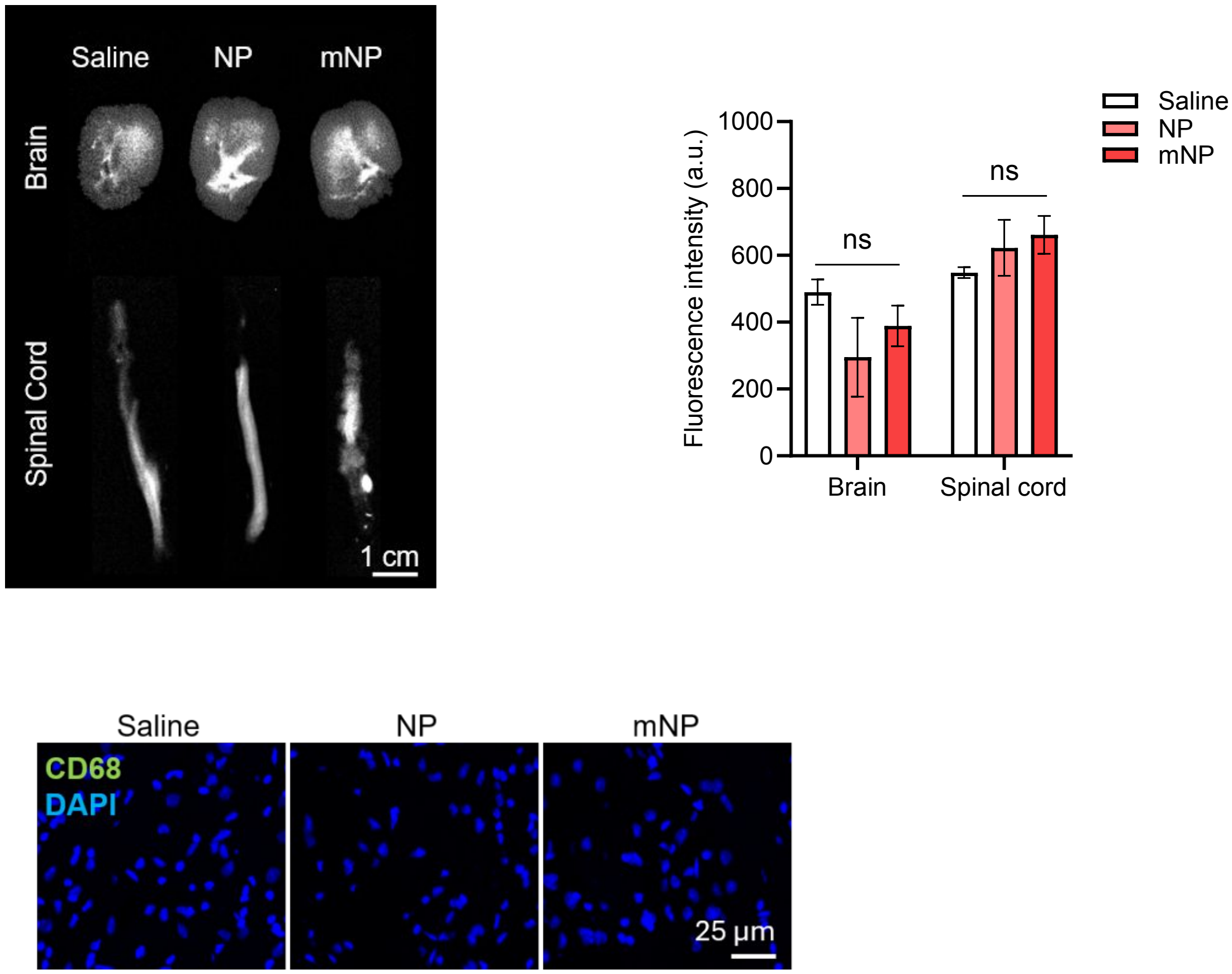

C.

Figure S2. Assessment of central nervous system accumulation of NP and mNP. Rats received a tail vein injection of saline, QD-NP or QD-mNP at EAN peak. After 24h, rats were perfused with saline, and brain and spinal cord were harvested, imaged *ex vivo*, and snap frozen. **(A)** Representative *ex vivo* images (Ex/Em=740/780 nm) and **(B)** quantified fluorescence in organ homogenate (Ex/Em=469/525 nm) demonstrate similar fluorescence levels in tissue from animals that received saline, NP, or mNP. **(C)** Sagittal sections of whole brain were labeled with CD68 primary and Alexa 488 secondary antibodies. No infiltration of macrophages or QD-NPs was detected. Data shown in B are the mean ± SEM, n = 3.

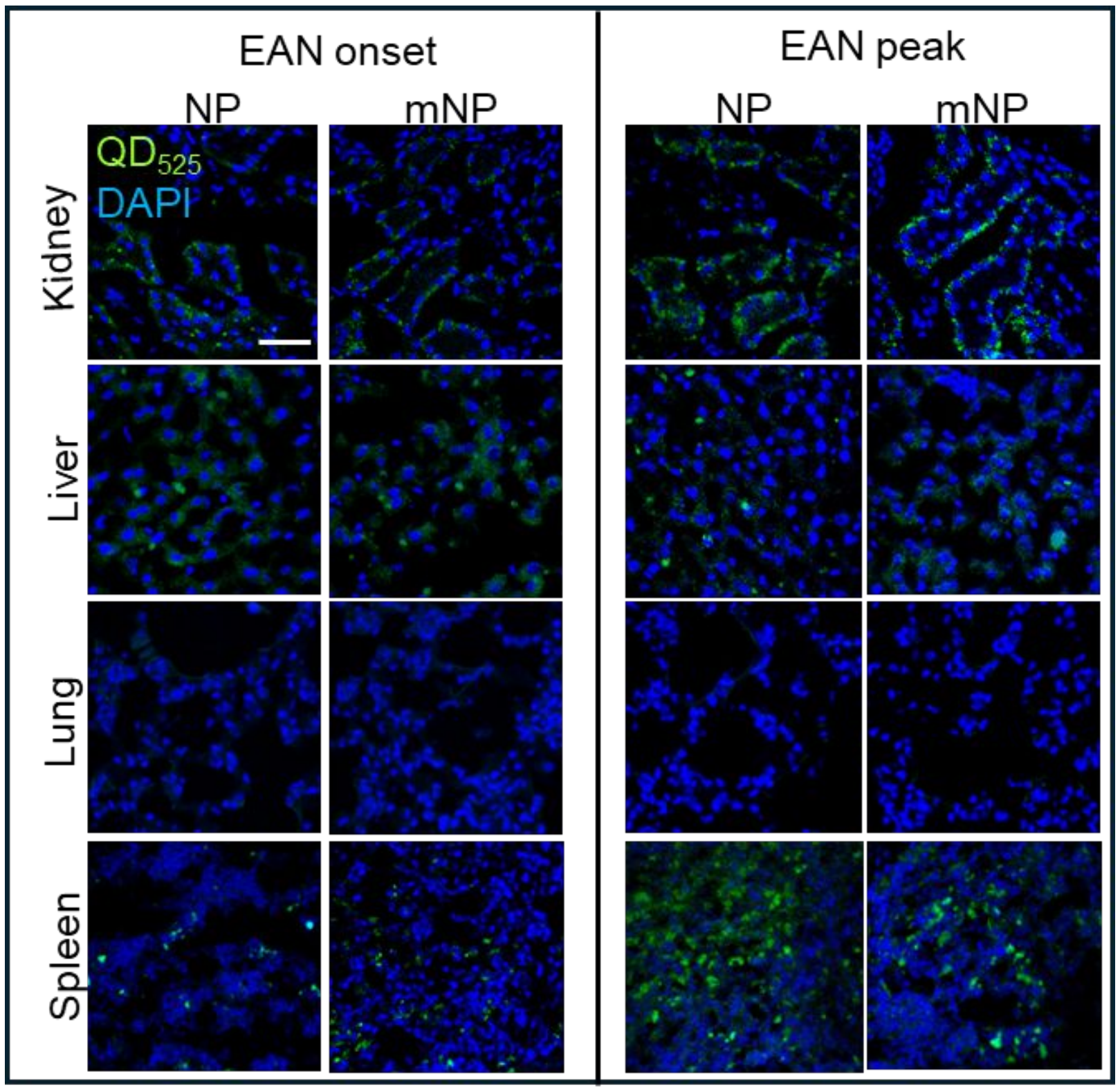

Figure S3. Assessment of NP and mNP accumulation in organs. Rats received a tail vein injection of saline, QD-NP or QD-mNP at EAN onset or peak. After 24h, rats were perfused with saline and kidney, liver, lung, and spleen were harvested and snap frozen. Representative confocal images demonstrate NP and mNP accumulation (green) in 10 µm cross sections from each organ. Images shown are representative of each group, Scale bar = 30 µm.

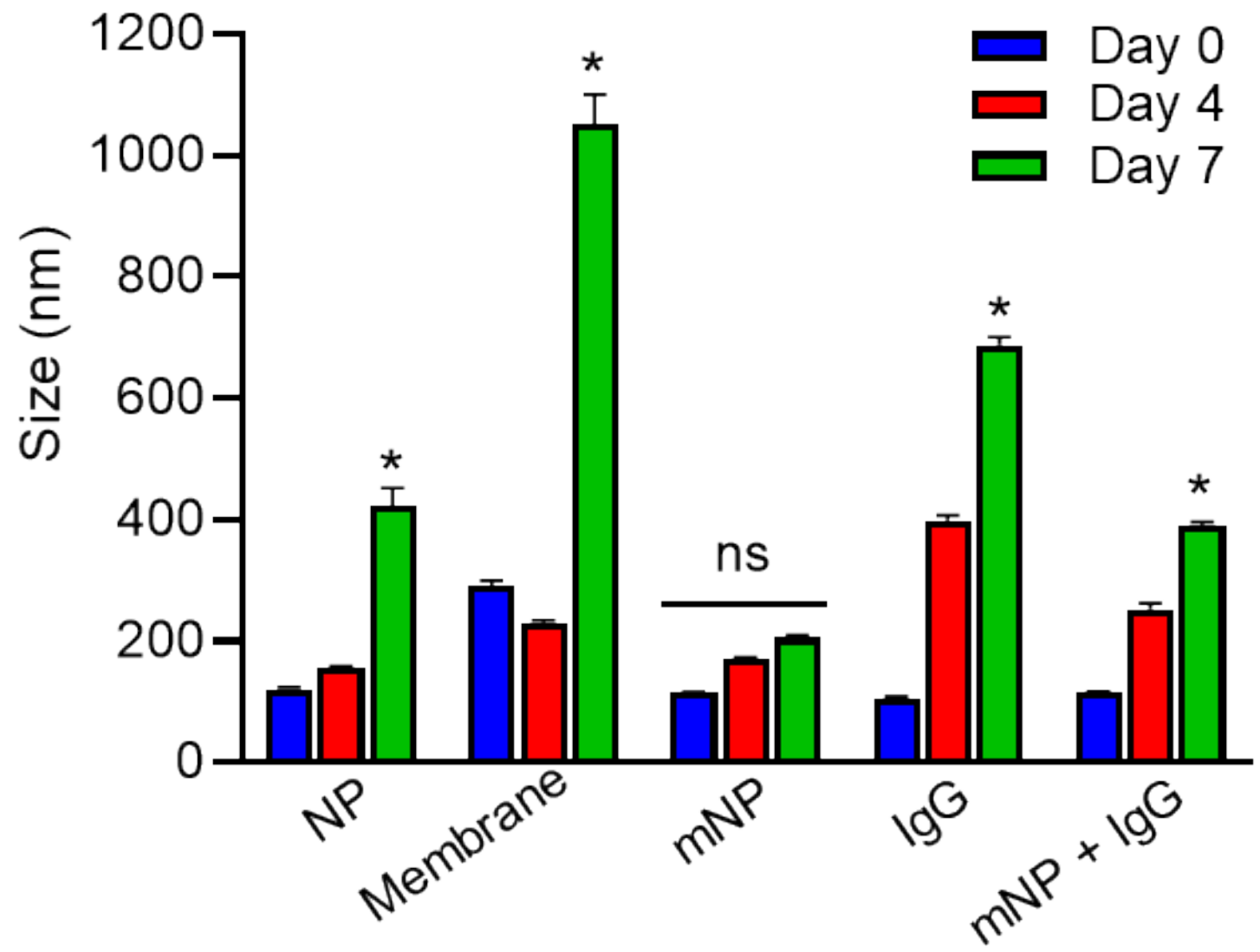

Figure S4. Translational *in vitro* assessment of component stability. Delivery system components and representative standard-of-care, as indicated, were suspended in 1x PBS and incubated at room temperature under constant agitation (360° rotation at 8 rpm). Size (nm) was assessed by dynamic light scattering on Day 0 (after suspension), Day 4, and Day 7. Data shown are the mean ± SEM, n = 3. *$p < 0.05$ Day 0 versus both Day 4 and Day 7 for the indicated conditions, two-way ANOVA with Tukey's post hoc test.
